# Automatic Diagnosis of Glaucoma from Retinal Images Using Deep Learning Approach

**DOI:** 10.3390/diagnostics13101738

**Published:** 2023-05-14

**Authors:** Ayesha Shoukat, Shahzad Akbar, Syed Ale Hassan, Sajid Iqbal, Abid Mehmood, Qazi Mudassar Ilyas

**Affiliations:** 1 Department of Computer Science, Riphah International University, Faisalabad Campus, Faisalabad 44000, Pakistan; 2 Department of Information Systems, College of Computer Sciences and Information Technology, King Faisal University, Al Ahsa 31982, Saudi Arabia; qilyas@kfu.edu.sa; 3 Department of Management Information Systems, College of Business Administration, King Faisal University, Al Ahsa 31982, Saudi Arabia

**Keywords:** glaucoma, fundus images, deep learning, early-stage detection, augmentation

## Abstract

Glaucoma is characterized by increased intraocular pressure and damage to the optic nerve, which may result in irreversible blindness. The drastic effects of this disease can be avoided if it is detected at an early stage. However, the condition is frequently detected at an advanced stage in the elderly population. Therefore, early-stage detection may save patients from irreversible vision loss. The manual assessment of glaucoma by ophthalmologists includes various skill-oriented, costly, and time-consuming methods. Several techniques are in experimental stages to detect early-stage glaucoma, but a definite diagnostic technique remains elusive. We present an automatic method based on deep learning that can detect early-stage glaucoma with very high accuracy. The detection technique involves the identification of patterns from the retinal images that are often overlooked by clinicians. The proposed approach uses the gray channels of fundus images and applies the data augmentation technique to create a large dataset of versatile fundus images to train the convolutional neural network model. Using the ResNet-50 architecture, the proposed approach achieved excellent results for detecting glaucoma on the G1020, RIM-ONE, ORIGA, and DRISHTI-GS datasets. We obtained a detection accuracy of 98.48%, a sensitivity of 99.30%, a specificity of 96.52%, an AUC of 97%, and an F1-score of 98% by using the proposed model on the G1020 dataset. The proposed model may help clinicians to diagnose early-stage glaucoma with very high accuracy for timely interventions.

## 1. Introduction

The major components of the human eye involved in vision are the cornea, pupil, iris, lens, retina, optic nerve, and tears [1]. The iris is located between the cornea and the lens and controls the light. The retina receives the light and transfers it to the brain for recognition by converting it into electrical signals. At the backside of the eye is a nerve known as the optic nerve, which comprises 1 million nerve fibers of the retinal ganglion cells [2]. The primary function of this nerve is to transfer visual signals from the retina to the occipital cortex.

**Figure 1:**
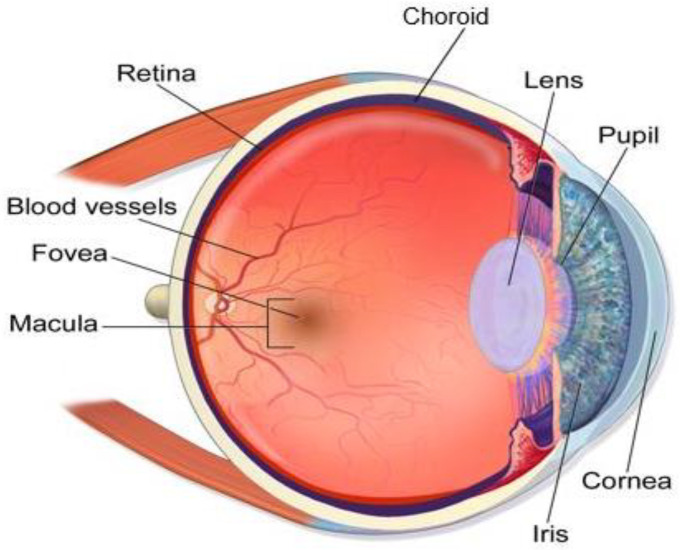
Anatomy of the human eye.

**Figure 2:**
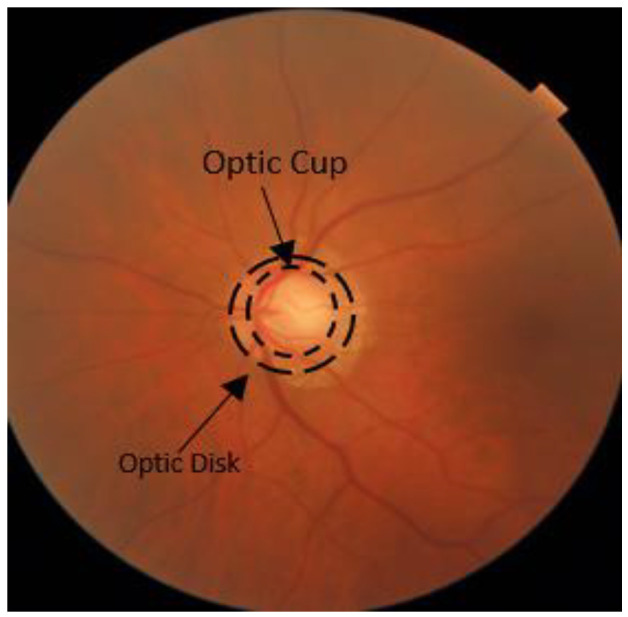
Enlarged optic cup within the optic disc in the glaucoma-affected image.

The human eye contains a fluid known as aqueous humor, which is continuously recycled. An obstruction in the drainage of aqueous humor leads to increased intraocular pressure (IOP). Consequently, the retina and optic nerve are damaged, which may lead to vision loss [3]. This is partly due to the degeneration of ganglion cells in the retina [2,4]. The loss of optic nerve fibers changes the shape of the optic disc (OD) towards an increase in the cup-to-disc ratio (CDR), which is an early sign of glaucoma [5]. The anatomy of the eye is depicted in Figure 1 [6]. The visual loss in glaucoma is due to damage to the retinal ganglionic cells [7,8]. The alterations in the visual field scope are essential for diagnosing glaucoma [6]. Figure 2 shows the enlarged CDR in an eye with glaucoma [5].

**Figure 3:**
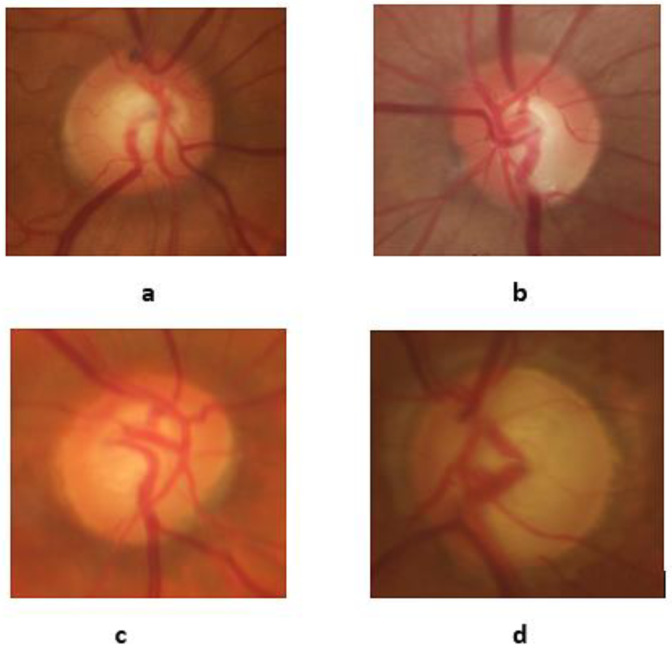
The fundus images of healthy control (**a**), early glaucoma (**b**), moderate glaucoma (**c**), and deep (advanced) glaucoma (**d**) from the RIM-ONE dataset.

Glaucoma is the second leading cause of blindness worldwide. About 80 million people [9] were affected by glaucoma worldwide in 2020, and the number may increase to 111.8 million by 2040 [10] There are several types of glaucoma, but the most common is open-angle glaucoma, which affects nearly 57.5 million people worldwide [10]. Regular checkups by ophthalmologists after age 50 can reduce the risk of developing glaucoma. Figure 3 shows the retinal fundus images of a healthy control and patients with early, moderate, and advanced-stage glaucoma from the RIM-ONE dataset [11].

Ophthalmologists use multiple manual methods to diagnose glaucoma, including gonioscopy, pachymetry, tonometry, and perimetry [12]. In tonometry, the IOP, a major risk factor for glaucoma, is measured. Gonioscopy measures the angle between the iris and cornea. Pachymetry measures the corneal thickness. However, these manual assessment methods for glaucoma detection are very time consuming and subjective. Further, they largely depend on the availability of ophthalmologists, which can be a limiting factor in remote areas. Therefore, there is currently a need for the development of automated tools that can efficiently diagnose glaucoma at an early stage.

Artificial intelligence technologies have grown significantly in recent years. Many efforts are being undertaken in healthcare to integrate AI technology for practical medical treatments [13,14,15]. Computer-aided diagnostic (CAD) tools for automatically detecting glaucoma are common in clinical practice. The applications of machine learning and, most recently, deep learning (DL) algorithms [16,17,18,19] have increased the diagnostic accuracy of these automated tools for detecting glaucoma.

Here, an efficient and fully automated system that is based on deep learning architecture and can efficiently diagnose early-stage glaucoma on given datasets is proposed. The following are the main contributions of this work:

- The most notable recent machine learning and deep learning-based glaucoma detection research is thoroughly reviewed to define the problem, focusing on various features that can support an efficient diagnosis.
- For the diagnosis, a model is developed employing advanced deep learning methods along with transfer learning, and the model is tuned using various techniques to lower the likelihood of model overfitting.
- Multiple datasets of glaucomatous retinal images are adopted to train and test the model to achieve higher diagnostic accuracy.
- An end-to-end learning system that overcomes the drawbacks of current glaucoma screening methods is developed.

The remaining part of the paper is organized into the following themes: The previous work by other researchers is explained in Section 2. Section 3 explains the proposed methodology. Section 4 describes the experimentation and results of the proposed model. Section 5 presents a discussion of the results, and Section 6 presents a conclusion summarizing the key findings.

## 2. Literature Review

Researchers have developed several techniques for the detection of glaucoma. Among these techniques, machine learning-based methods [20] manually extract the features and perform classification by using different machine learning classifiers. Recently, deep learning models, such as convolutional neural networks (CNNs), have been widely used to diagnose diseases automatically without human involvement. Glaucoma detection through CNNs is performed by various researchers [21,22,23,24,25,26,27,28,29,30]. The CNN-based systems perform effective computation and provide robust results for disease classification. A CNN consists of different layers, such as convolutional, activation, pooling, and the fully connected layer (FCL). Each architecture consists of a different combination of these layers. The diagnosis and detection of other retinal diseases such as papilledema [23,31], diabetic retinopathy [23], central serous retinopathy (CSR) [32,33], and hypertensive retinopathy [22] can be performed through deep learning and machine learning methodologies using OCT and fundus images [5,30]. Diabetes and other eye diseases have been successfully diagnosed by DL techniques [23].

The application of the CAD system has widened the diagnostic horizon in several other disease diagnoses, such as CSR [33], lung tumor [34], brain tumor [35], skin tumor [17], and prostate cancer [18]. The fundus images provide a clear picture of the eye’s internal structure and are widely used for glaucoma diagnosis. The glaucoma classification using fundus images through DL models has shown encouraging results [36,37]. The fundus images clearly depict the optic nerve head and are readily available for training the glaucoma detection models [38]. Various models based on pre-trained CNN models [14,39], ensemble approaches [40,41,42] and CNN-based architectures are encountered in this article for the detection of glaucoma.

Serte and Serener developed a glaucoma detection model using an ensemble approach based on a local dataset of 1542 fundus images [40]. The model cropped the OD by using a graph saliency region technique. Three CNN architectures, namely ResNet-50, ResNet-152, and AlexNet, were used as the ensemble classifiers in this model. All three methods, including without saliency map, with saliency map and single CNN model, and with saliency map and ensemble approach, were tested, and the best results were obtained for the ensemble approach with an AUC of 94% and accuracy of 88%. Chaudhary and Pachori developed a glaucoma detection model based on two methods, using RIM-ONE, ORIGA, and DRISHTI-GS datasets [41]. The 2D Fourier–Bessel series expansion-based empirical wavelet transform was used for the segmentation of the boundary. Two methods were used, one depending on the ML model and the other using the ensemble approach of the CNN architecture ResNet. The first model at full scale obtained the best results. The best results with the second method were obtained with the ensemble technique at a full scale with 91.1% accuracy, 91.1% sensitivity, 94.3% specificity, 83.3% AUC, and 96% ROC. GlaucomaNet [42] was proposed to identify POAG based on dataset images from different populations and settings. The model comprises two CNNs intended to mimic the human grading process. To this end, the first CNN learns the discriminative features, whereas the second fuses the features for grading. This simulation of the human grading process combined with an ensemble of network architectures greatly enhanced the diagnostic accuracy.

Thakoor et al. developed a model based on different CNN architectures trained on OCT images and also used some pre-trained models to detect glaucoma [14]. The pre-trained ResNet, VGG, and InceptionNet were combined with random forest and compared with the CNN architectures trained on OCT images. A high accuracy of 96.27% was achieved with the CNN trained on the OCT images. Hemelings et al. proposed an approach for glaucoma detection using pre-trained ResNet-128 architecture with 7083 OD center fundus images [39]. The transfer and active learning approaches were used to enhance the diagnosis capability of the model. The use of a saliency map highlighted the affected region to provide evidence of the disease. The model achieved robust results with an AUC of 99.55%, a specificity of 93%, and a sensitivity of 99.2% for glaucoma detection.

Yu et al. [4] developed a model using a modified version of U-Net architecture in fundal images for glaucoma diagnosis using multiple datasets. The U-Net used the pre-trained ResNet-34 as an encoder and the classical U-Net architecture as a decoder. The model showed good performance as 97.38% of disc dice values and 88.77% of cup dice values were aligned with the DRISHTI-GS test set. Other authors proposed an approach named AG-CNN, which detected glaucoma and localization of pathological areas using the fundus images [6]. The model is based on attention prediction, localization of the affected area, and glaucoma classification. The deep features predicted glaucoma through the visual maps of necrotic areas in the LAG and RIM-ONE datasets. The use of attention maps for localizing the pathological area demonstrated high efficacy. The model prediction for glaucoma was superior to previous models, with an accuracy of 95.3%. 

Phan et al. developed a model based on three CNN architectures, ResNet-152, VGG19, and DenseNet201, for diagnosing glaucoma on 3312 retinal fundus images [25]. The proposed model has also been tested on poor-quality images to examine its diagnostic accuracy in glaucoma. All the architectures achieved an AUC of 90% for detecting glaucoma. Liao et al. [43] proposed a novel CNN-based scheme that used ResBlock architecture to diagnose glaucoma using the ORIGA dataset. The model diagnosed glaucoma and provided a transparent interpretation based on visual evidence by highlighting the affected area. The model named EAMNet contained three parts: ResNet architecture extracted the features and aggregation, and the multiple-layer average pooling (M-LAP) linked the semantic detail and information of the localization, while the evidence activation map (EAP) was used for the evidence of the affected area the physician used for the final decision. The activation map was used to provide the clinical basis for glaucoma. The proposed scheme efficiently diagnosed glaucoma, with an AUC of 0.88.

Researchers developed the G-Net model based on CNN to detect glaucoma in the DRISHTI-GS dataset [44]. The model used two neural networks (U-Net) to separate the disc and cup. The cropped fundus images in the red channel were fed to the model. The model contained 31 layers of convolutional, max-pooling, up-sampling, and merge layers. The filters applied were of sizes (3, 3), (1, 1), and (1, 32), and 64 filters were used on different layers. The model labeled the pixel as black on segmenting the OD in the real image and white otherwise. The output images were fed to the other model to segment the cup. The second model was like the first model, with a single difference in the size of the filters (4, 4). The output of this model was a segmented cup. These two outputs were used to calculate the CDR for the glaucoma prediction. This algorithm used two neural networks to obtain a high accuracy of 95.8% for OD and 93.0% for OC segmentation.

Researchers developed a model based on CNN for glaucoma detection using 1110 OCT images and compared its performance with the ML algorithms [45]. A total of 22 features were extracted and fed to different machine learning classifiers such as NB, RF, SVM, LR, Gradient Adaboost, and Extra Trees. The CNN model classified and achieved better results with an AUC of 0.97 than other machine learning approaches, such as logistic regression, with an AUC of 0.89.

Thakur et al. proposed a model capable of diagnosing glaucoma before the onset of the disease [46]. Three deep learning models were trained on 66,721 fundus images that can detect glaucoma, such as 1 to 3 years ago, 4 to 7 years ago, and before the onset of glaucoma. All three models achieved AUCs of 0.88, 0.77, and 0.97 in detecting glaucoma. Lima et al. developed a CNN model for the optic cup segmentation for the detection of glaucoma [47]. The modified U-Net architecture segmented the optic cup from the green channel image, and the optic disc mask was given as input. The model achieved a dice value of 94% on the DRISHTI dataset.

Maheshwari et al. presented a model that converted the images into RGB channels after dividing the dataset images into training and testing images [15]. The LBP-based augmentation was applied to obtain the best results. The model achieved 98.90% accuracy, 100% sensitivity, and 97.50% specificity. Lima et al. used a genetic model based on CNN with 25 layers using the RIM-ONE dataset to diagnose glaucoma [12]. The model achieved an accuracy of 91% in detecting glaucoma. Saxena et al. developed a six-layer CNN model for glaucoma detection using the SCES and the ORIGA datasets [13]. The ROI was extracted using the ARGALI approach, and the data augmentation technique was used to avoid the overfitting problem. The model achieved excellent results, with an AUC of 0.882 on SCES and 0.822 on ORIGA datasets. Elangovan and Nath developed a CNN-based model consisting of 18 layers for glaucoma detection [48]. The model was based on DRISHTI–GS1, ORIGA, RIM–ONE2 (release 2), ACRIMA, and LAG datasets. The best results were obtained with the ACRIMA dataset, achieving 96.64% accuracy, 96.07% sensitivity, 97.39% specificity, and 97.74% precision. Aamir et al. [49] developed a multi-level CNN model for diagnosing glaucoma. The fundus images were preprocessed to reduce noise with the adaptive histogram equalizer technique. The model classified the fundus images for glaucoma detection into advanced, moderate, and early categories. The model achieved a sensitivity of 97.04%, a specificity of 98.99%, an accuracy of 99.39%, and a PRC of 98.2% on 1338 fundus images. Raja et al. [50] proposed a technique for diagnosing glaucoma using a dataset of 196 OCT images. The proposed model used CNN and calculated the CDR with 94% accuracy, 94.4% sensitivity, and 93.75% specificity in detecting glaucoma. Carvalho et al. [51] proposed a 3DCNN algorithm for diagnosing glaucoma through the fundus images of RIM-ONE and DRISHTI-GS datasets. The 2D fundus images were converted into 3D volumes for each RGB and gray channel. The CNN was trained on all four channels and showed the best results on a gray channel with 83.23% accuracy, 85.54% sensitivity, 80.95% specificity, 83.2% AUC, and 66.45 Kappa.

Gheisari et al. developed a combined model based on a CNN and a recurrent neural network for diagnosing glaucoma using retinal fundus images [52]. The diagnostic results were achieved with an F-measure of 96.2% on 295 videos and 1810 fundus images. Veena et al. developed a CNN model for the detection of glaucoma [53]. The images were preprocessed to eliminate the noise using the Gaussian filter. The Sobel edge and the watershed algorithms extracted the features from the fundus images. The model achieved the OD and OC segmentation accuracies of 0.9845 and 0.9732, respectively, on the DRISHTI dataset. The achieved results are 98.48% accuracy, 99.3% sensitivity, 96.52% specificity, 97% AUC, and 98% of F1-score on the G1020 dataset.

Recently, Fan et al. [54] assessed the diagnostic precision, generalizability, and explainability of a Vision Transformer deep learning method in diagnosing the primary open-angle glaucoma and identifying the salient areas found in the retinal images. A dual learning-based technique that combines deep learning and machine learning was proposed by Thanki [55]. For identifying distinctive retinal characteristics, a deep neural network extracts deep features. Following that, a hybrid classification algorithm is employed to accurately classify glaucomatous retinal images. The following Table 1 shows the summary of year-wise published studies for the detection of glaucoma.

## 3. Proposed Methodology

**Figure 4:**
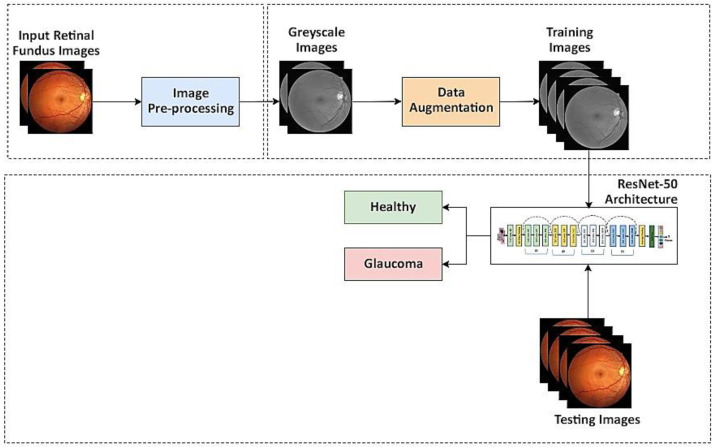
Flow diagram of the proposed model for glaucoma detection.

The innovation in artificial intelligence may help in a fast and accurate diagnosis of diseases. The proposed model is developed using the ResNet-50 robust image classification architecture. The fundus imaging modality is used as it precisely depicts the eye’s internal structure. The applications of fundus images are numerous for many other disease diagnoses, such as cataracts, retinopathy of prematurity, DR, and age-related macular degeneration (AMD) [56]. Figure 4 depicts the flow diagram of the proposed model.

### 3.1. Dataset

In this research, four publicly available datasets are used for testing and training the model: (i) G1020 [57], (ii) DRISHTI-GS [58], (iii) RIM-ONE [11], and (iv) ORIGA [59]. The G1020 dataset comprises 1020 fundus images with high resolution, CDR calculation, OD and OC segmentation, size of the neuro-retinal rim in inferior, superior, nasal, and temporal regions, and location of the bounding box for OD for glaucoma detection. The images in the dataset are only focused on the fundus region by removing the unrelated region. The size of the images is between 1944 × 2108 and 2426 × 3007 pixels. The dataset is publicly available.

The DRISHTI-GS dataset contains OD and OC segmented and ground truth images. This dataset contains 101 images, of which 31 are healthy and 70 include eyes with glaucoma. The fundus images in this dataset are focused on the OD with a field of view of 30 degrees, and the image resolution is 2896 × 1944 pixels in PNG format. Six experts performed the manual annotation of OD and OC in this dataset. This dataset is publicly available. The RIM-ONE dataset comprises 169 ONH segmented high-resolution fundus images. The images are captured with a fundus camera (Nidek AFC-210). There are four categories of images, including 118 normal, 12 early glaucoma, 14 moderate, 14 deep, and 11 images for ocular hypertension. These images are also publicly available. The ORIGA dataset comprises 650 segmented and annotated images. Every image is labeled with grading information. This dataset can be used for image processing algorithms and the method for detection of peripapillary atrophy (PPA) detection and the junction of the disc boundary blood vessels.

### 3.2. Image Preprocessing

**Figure 5:**
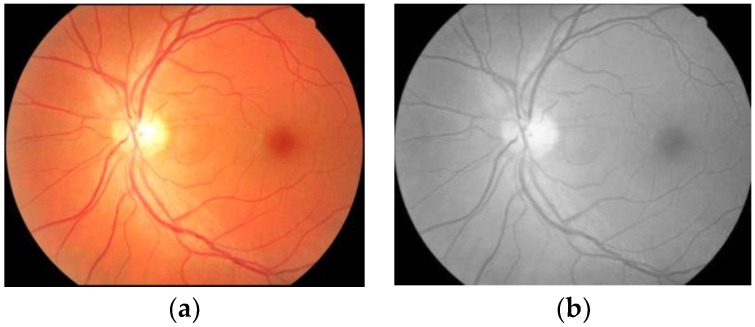
Fundus image with grayscale conversion (**a**) Normal fundus image (**b**) Greyscale fundus image.

Image preprocessing is performed to expand the image quality for further analysis. This often helps to produce more robust results from the CNN architecture. The greyscale images are obtained from all the training images collected from the G1020, DRISHTI-GS, ORIGA, and RIM-ONE datasets. The grayscale image modality provides a clear and sharper view of the fundus images, as displayed in Figure 5 [57]. The grayscale morphology synthesizes all pixels with a homogeneous intensity value. All the training images are converted into gray channels. The OD-centered images in grayscale are fed to the ResNet-50 model for training.

### 3.3. Data Augmentation

The data augmentation technique has been used to increase the number of images when the available data are inadequate for statistical and biological significance. The augmentation technique is a better approach to overcome this problem due to the limited availability of images in the medical field. This technique slightly modifies the existing data to create more copies of the data. The data augmentation technique can also overcome the overfitting of the deep learning models by enhancing the model’s performance and diagnostic capability. Different techniques are applied, such as flipping the images horizontally and vertically, rotation, cropping, and scaling.

### 3.4. Transfer Learning

The deep learning model’s training from scratch is tedious work requiring a large image dataset and efficient hardware. Additionally, it also requires more training time. The transfer learning approach uses the pre-trained model, which is trained on a large number of images such as ImageNet [60]. It transfers the knowledge learned from the model to another model even if the field is different [61]. The pre-trained model is trained according to the new data by changing some parameters. In this work, the pre-trained CNN architecture ResNet-50 is retrained on the G1020, ORIGA, RIM-ONE, and DRISHTI-GS datasets.

### 3.5. Convolutional Neural Network

The CNN is a multilayer DL network that obtains the input as high-dimension data (images) and progressively extracts high-dimension features from the input images. The CNN architectures consist of different numbers of layers, which increase as the size of the input images increases. The network learns more accurately as it goes deeper. However, the major drawback of the deeper networks is the increase in computation time. CNNs have shown promising image processing, object detection, image segmentation, image classification, video processing, and natural language processing features [61]. The applications of CNN architectures have shown tremendous results for disease diagnosis in the medical sciences. 

### 3.6. ResNet-50 Architecture

**Figure 6:**
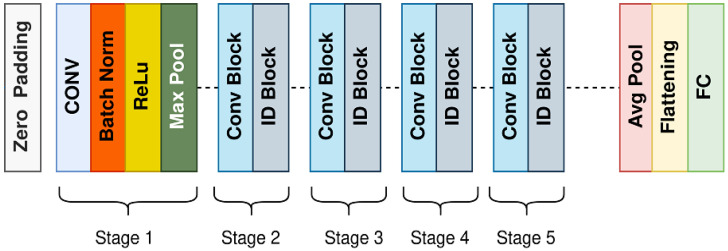
Block diagram of ResNet-50 architecture.

The ResNet is the short form of the residual network, and it solves the vanishing gradient problem by using the skip connection approach. Before ResNet, network degradation problems occurred due to increased network depth. The result of this degradation was a higher training error. To overcome this problem, the skip connection technique is applied in the ResNet architecture. This architecture shows higher detection accuracy, takes less training time, and is easier to optimize. The ResNet architecture has several applications for image processing and diagnosis of diseases in the medical field. Additionally, it has shown excellent results for object detection and face recognition. Figure 6 shows the architecture of ResNet-50 [62]. The difference in the ResNet-50 from the earlier ResNet-18 and ResNet-34 is skipping three layers instead of two and using a 1 × 1 convolution layer. There are 50 layers in this architecture, and it is capable of classifying data into seven classes. It is widely applied for image recognition, object localization, and object detection. Consequently, it has considerably reduced computational costs [63]. 

The block diagram of the proposed methodology for glaucoma detection is shown in Figure 4. The proposed methodology consists of the following steps:

- Acquire the fundus images from different publicly available datasets.
- Convert the fundus images into grayscale.
- Apply the data augmentation approach to multiply the number of images by flipping, rescaling, and rotation after dividing the dataset into training and testing sets. Further, 80% of the images in the dataset are used for training, 10% of images for validation, and the remaining 10% for testing.
- Pre-trained DL architecture, such as the ResNet-50, is used for classification.
- The model classifies an image as either a healthy or glaucomatous image.

## 4. Experiments and Results

The proposed model is evaluated using performance metrics such as accuracy, sensitivity, and specificity. There are four possibilities for the classified images, namely true positive, true negative, false positive, and false negative. The true positive labels the image as affected by glaucoma, and it is a correct prediction. The true negative labels the image as a healthy image, and it is also correctly classified. The false positive erroneously labels an image as a glaucoma-affected, otherwise healthy image. The false negative incorrectly labels a glaucoma-affected image as a healthy image.

The accuracy is the measure of the correctly labeled images divided by the total number of images. It can be calculated as in Equation (1).

(1) $$Accuracy=\frac{TP+TN}{TP+TN+FP+FN}$$

The sensitivity represents the correctly classified images affected by glaucoma. It is calculated as in Equation (2):

(2) $$Senstivity=\frac{TP}{TP+FN}$$

The specificity represents the correctly classified healthy images. It can be calculated as Equation (3):

(3) $$Specificity=\frac{TN}{TN+FP}$$

The F1-score can be calculated as in Equation (4):

(4) $$F1-Score=\frac{2TP}{2TP+FP+FN}$$

**Figure 7:**
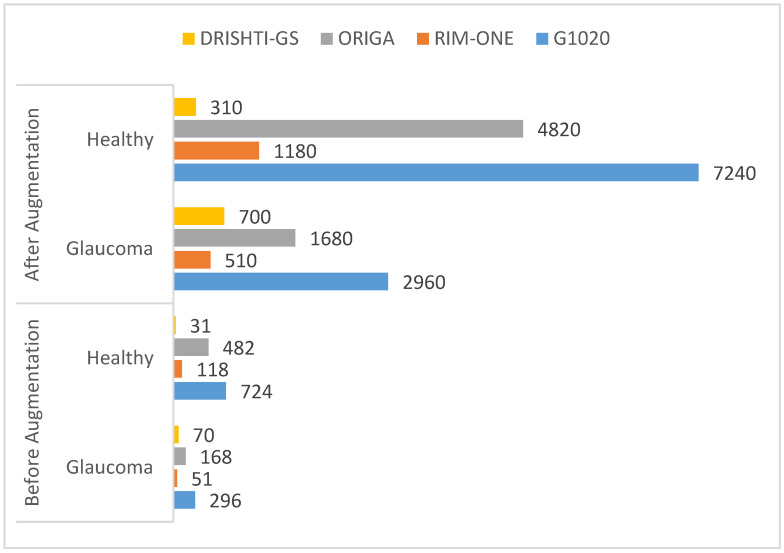
Dataset comparison before and after data augmentation.

**Figure 8:**
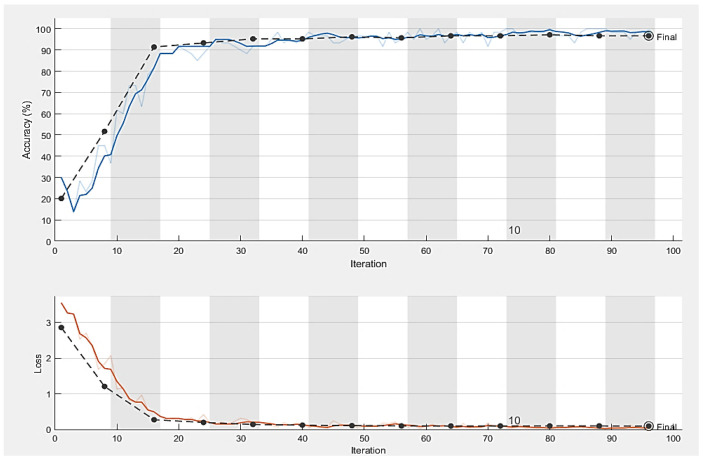
Training process curve of G1020 dataset.

**Figure 9:**
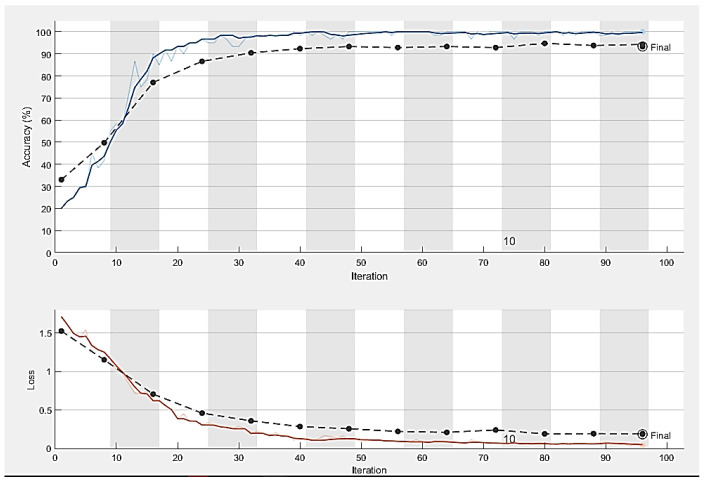
Training process curve of the DRISHTI-GS dataset.

**Figure 10:**
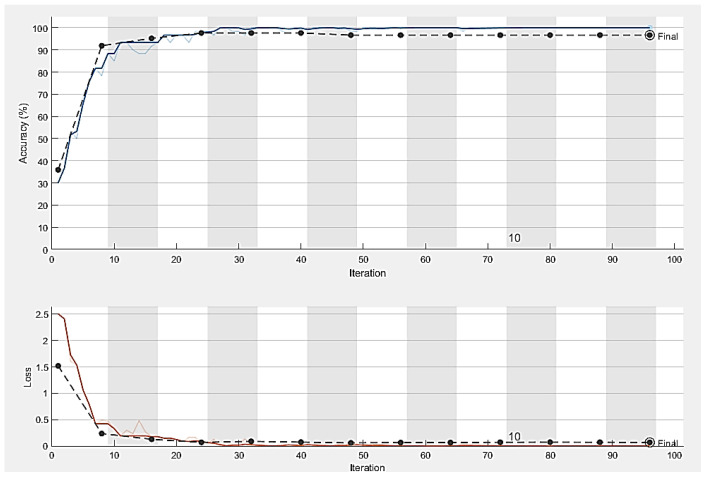
Training process curve of RIM-ONE dataset.

**Figure 11:**
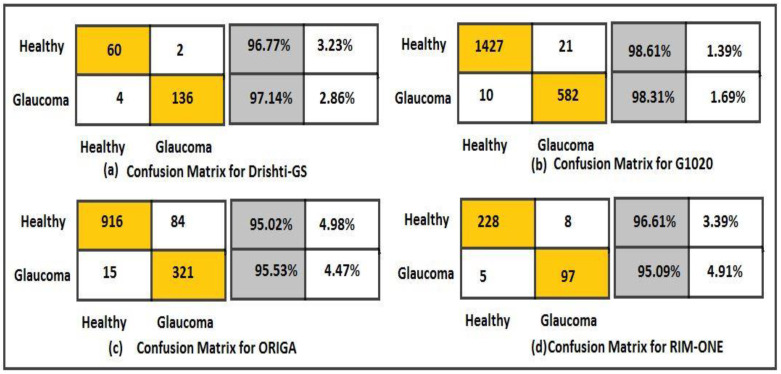
Confusion matrix for data validation on (**a**) DRISHTI-GS, (**b**) G1020, (**c**) ORIGA, and (**d**) RIM-ONE datasets.

The dataset’s fundus images were divided into three subcategories: training, validation, and testing. We used 80% of the images for the training of the model, 10% for model validation, and the remaining 10% for testing. All the images were resized to the same size and centered on the optic disc. Moreover, the model was trained using the SDG solver with a learning rate of 0.001 on ten epochs in Python with a system configuration of Intel/Xeon/CPU E3-1225, 3.3 GHz, and 16 GB RAM. The computational time for training of model on these datasets was 30 min. Figure 7 shows the number of images before and after data augmentation. Four datasets, namely G1020, RIM-ONE, ORIGA, and DRISHTI-GS, were used. The ResNet-50 architecture achieved robust results with 98.48% accuracy, 96.52% specificity, 99.30% sensitivity, 97% AUC, and an F1-score of 98% on the G1020 dataset. The comparison of the proposed model with the previous studies is shown in Table 2. Figure 8, Figure 9 and Figure 10 show the accuracy and error rate for the training data using ResNet-50 over G1020, DRISHTI-GS, RIM-ONE, and ORIGA datasets, respectively. Figure 11 shows the confusion matrix of validation data of all four datasets.

## 5. Discussion

The proposed model uses the deep learning architecture ResNet-50 to diagnose early-stage glaucoma using fundus images. Four datasets, G1020, DRISHTI-GS, RIM-ONE, and ORIGA, were used for the proposed model’s training, validation, and testing. The capability of deep learning models for automatic identification of the pattern from images has smoothed the data for obtaining robust results for disease detection. The greater number of layers in the model requires more training time, and sometimes deeper models take several weeks for training, which is not optimal in clinical settings. The pre-trained ResNet-50 architecture can best classify the images in reduced computation time. The training of the model from scratch requires a large amount of data and training time. So, the transfer learning approach is applied to save computation time and achieve robust diagnostic results. The use of pre-trained models while training the CNN architectures for a new task has made it possible to develop a fast and reliable diagnosis system, despite the limited availability of the required data. The dataset’s limited images cause overfitting of the model, but the data augmentation technique overcomes this problem. Many images can be created from a single image, providing a large dataset for the DL models for training.

The fundus images are a cheap solution for the diagnosis of glaucoma. The fundus images in the gray channel depict the lesion more precisely and clearly indicate the affected region. The G1020, RIM-ONE, ORIGA, and DRISHTI-GS datasets, which contain the OD segmented images, were applied. The proposed model has exhibited glaucoma detection with 98.48% accuracy, 99.30% sensitivity, 96.52% specificity, an AUC of 97%, and an F1-score of 98% on the G1020 dataset. The proposed model’s results on the ORIGA dataset include 92.59% accuracy, 98.39% sensitivity, 79.26% specificity, 93% AUC, and 95% F1-score. The RIM-ONE dataset has shown 96.15% accuracy, 97.85% sensitivity, 92.38% specificity, 94.2% AUC, and 97% F1-score on the proposed model. The DRISHTI-GS has shown 97.03% accuracy, 93.75% sensitivity, 98.55% specificity, 96% AUC, and 97% F1-score. The results of the proposed model on all four datasets are shown in Table 2.

The proposed model has shown more robust results than the existing techniques on the G1020 dataset. Due to the wide availability of high-resolution images in the G1020 dataset, the best performance of the proposed model is obtained on the G1020 dataset. The performance of the proposed model is poor on the ORIGA dataset compared to other datasets in terms of specificity. This is due to poor preprocessing results on the images of the ORIGA dataset through the proposed technique.

## 6. Conclusions

Glaucoma can severely damage the eyes and leads to irreversible vision loss if left untreated. Several methods are developed for glaucoma diagnosis using various approaches. The proposed model has used four different datasets and shows high efficacy for diagnosing glaucoma at an early stage using the gray channel of fundus images. The model uses the data augmentation technique to provide a wide variety of fundus images for the training. The proposed model has shown 98.48% accuracy, 99.30% sensitivity, 96.52% specificity, AUC of 97%, and an F1-score of 98% on the G1020 dataset with the ResNet-50 architecture. The self-interpretation of CNN architectures to detect the abnormalities for disease diagnosis may assist clinicians in the timely diagnosis and treatment of glaucoma. In the future, new models based on both the fundus and the OCT images can be developed to diagnose early-stage glaucoma using a multimodal imaging approach. 

## Figures and Tables

**Table 1:** Summary of Literature Review.

Sr. No.	Authors	Year	Model	Datasets	Results
1	Yu et al. [4]	2019	Pre-trained U-Net, ResNet	RIGA, DRISHTI-GS, RIM-ONE	Dice 97.38% (Disc) Dice 88.77% (Cup)
2	Li et al. [6]	2019	CNN	LAG, RIM-ONE	Accuracy 95.3%
3	Phan et al. [25]	2019	ResNet-152, DenseNet201, VGG19	Local dataset of 3777 images	AUC 0.9
4	Liao et al. [43]	2019	ResNet	ORIGA	Accuracy 0.88
5	Serte et al. [40]	2019	ResNet-50, ResNet-152, and GoogleNet (ensemble method)	HRF, DRISHTI-GS1, RIMONE, sjchoi86-HRF, ACRIMA	Accuracy 53%, AUC 83%, specificity 100%
6	Juneja et al. [44]	2019	U-Net	DRISHTI-GS	Accuracy 95.8% (OD segmentation), 93.0% (OC segmentation)
7	Maetschke et al. [45]	2019	CNN	Local dataset of 1110 images	AUC 0.94
8	Thakoor et al. [14]	2019	Pre-trained CNN	Local dataset of 737 images	Accuracy 96.27%
9	Maheshwari et al. [15]	2020	AlexNet	RIM-ONE	Accuracy: 98.90% Sensitivity: 100% Specificity: 97.50%
10	Lima et al. [12]	2020	CNN	RIM-ONE r3	Accuracy 91%
11	Saxena et al. [13]	2020	CNN	ORIGA, SCES	AUC 0.822 (ORIGA) AUC 0.882 (SCES)
12	Thakur et al. [46]	2020	MobileNet v2	Local datasets of 45,301, 42,601, and 42,498 images	AUC 0.97
13	Hemelings et al. [39]	2020	Pre-trained ResNet 128	Local dataset of 1424 images	AUC 0.995 Sensitivity 99.2% Specificity 93%
14	Elangovan and Nath [48]	2020	CNN	RIM-ONE, DRISHTI–GS1, ORIGA, LAG, ACRIMA	Accuracy 96.64%, sensitivity 96.07%, specificity 97.39%, precision 97.74%
15	Aamir et al. [49]	2020	ML-DCNN	Local dataset of 1338 fundus images	Sensitivity 97.04%, specificity 98.99%, accuracy 99.39%, PRC 98.2%
16	Raja et al. [50]	2020	CNN	Local dataset of 196 OCT images	Accuracy 94%, sensitivity 94.4%, specificity 93.75%
17	Gheisari et al. [52]	2021	CNN, RNN	295 videos and local dataset of 1810 fundus images	F-measure 96.2%
18	Chaudhary and Pachori [41]	2021	Ensemble ResNet Models	RIM-ONE, ORIGA, and DRISHTI-GS	Accuracy 91.1%, sensitivity 91.1%, specificity 94.3%, AUC 83.3%, ROC 96%
19	Carvalho et al. [51]	2021	3DCNN	RIM-ONE and DRISHTI-GS	Accuracy 83.23%, sensitivity 85.54%, specificity 80.95%, AUC 83.2%, and Kappa 66.45%
20	Lin et al. [42]	2022	CNN	OHTS and LAG	Accuracy 0.930 (OHTS) and 0.969 (LAG)
21	Veena et al. [53]	2022	CNN	DRISHTI-GS	Accuracy 98% (OD), 97% (OC)
22	Fan et al. [54]	2023	CNN	Custom assembled from 5 public datasets	AUC 0.91
23	Thanki [55]	2023	Deep NN	DRISHTI-GS and ORIGA	Accuracy 100%

**Table 2:** Comparison Table of the Proposed Model with State-of-the-Art Models.

Sr #	Authors	Dataset	AUC	Accuracy	Sensitivity	Specificity	F1-Score
1	Lima et al. [12]	RIM-ONE r3	91%	**-**	**-**	**-**	**-**
2	Saxena et al. [13]	SCES	88.2%	**-**	**-**	**-**	**-**
3	Thakoor et al. [14]	Local dataset of 737 images	**-**	96.27%	**-**	**-**	**-**
	Fan et al. [54]	OHTS	-	91%	**-**	**-**	-
		DIGS		74%			
		ACRIMA		74%			
		LAG		79%			
		RIM-ONE		90%			
		ORIGA		55%			
	Lin et al. [42]	OHTS LAG	90.4%	93%			49%
	Thanki [49]	ORIGA	69.7%	76.2%	100%		73%
	Veena et al. [53]	DRISHTI–GS		98%			95.41%
4	Gomez-Valverde et al. [64]	Local dataset of 2313 images	94%	87.01%	89.01%	89.01%	-
5	Christopher et al. [65]	Local dataset of 14,822 images	97%	88%	95%	95%	-
6	Thakur et al. [46]	Local datasets of 45,301, 42,601, and 42,498 images	97%	**-**	**-**	**-**	**-**
**Proposed** **Method**		**RIM-ONE**	94.2%	96.15%	97.85%	92.38%	97%
		**ORIGA**	93%	92.59%	98.39%	79.26%	95%
		**G1020**	97%	98.48%	99.30%	96.52%	98%
		**DRISHTI-GS**	96%	97.03%	93.75%	98.55%	97%

## Data Availability

The data that support the findings of this study are openly available in the following repositories: https://www.kaggle.com/datasets/arnavjain1/glaucoma-datasets?select=G1020. https://www.kaggle.com/datasets/lokeshsaipureddi/drishtigs-retina-dataset-for-onh-segmentation. https://www.kaggle.com/datasets/lucascunhadecarvalho/rimoner2. https://www.kaggle.com/datasets/arnavjain1/glaucoma-datasets?select=ORIGA.
